# Identification of a pyroptosis-related prognosis gene signature and its relationship with an immune microenvironment in gliomas

**DOI:** 10.1097/MD.0000000000029391

**Published:** 2022-07-15

**Authors:** Shengying Xiao, Zhiguang Yan, Furen Zeng, Yichen Lu, Jun Qiu, Xiaodong Zhu

**Affiliations:** a Department of Radiation Oncology, Guangxi Medical University Cancer Hospital, Nanning, Guangxi 530021, P.R. China; b Department of Oncology, Hunan Provincial People’s Hospital (The First Affiliated Hospital of Hunan Normal University), Changsha, Hunan 410016, P.R. China; c Department of Orthopedics, Ningxiang Hospital Affiliated to Hunan University of Chinese Medicine, Ningxiang, Hunan, 410600, P.R. China.

**Keywords:** pyroptosis-related genes, prognosis, bioinformatics, immunity

## Abstract

**Background:**

Glioma is the most common type of primary brain cancer, and the prognosis of most patients with glioma is poor. Pyroptosis is a newly discovered inflammatory programmed cell death. However, the expression of pyroptosis-related genes (PRGs) in glioma and its correlation with prognosis are unclear.

**Methods:**

27 pyroptosis genes differentially expressed between glioma and adjacent normal tissues were identified. All glioma cases could be stratified into 2 subtypes based on these differentially expressed PRGs. The prognostic value of each PRG was evaluated to construct a prognostic model.

**Results:**

A novel 16-gene signature was constructed by using the least absolute shrinkage and selection operator Cox regression method. Then, patients with glioma were divided into low- and high-risk groups in the TCGA cohort. The survival rate of patients in the low-risk group was significantly higher than that in the high-risk group (*P* = .001). Patients with glioma from the Gene Expression Omnibus (GEO) cohort were stratified into 2 risk groups by using the median risk score. The overall survival (OS) of the low-risk group was longer than that of the high-risk group (*P* = .001). The risk score was considered an independent prognostic factor of the OS of patients with glioma. Gene ontology and Kyoto Encylopedia of Genes and Genomes analysis showed that the differentially expressed PRGs were mainly related to neutrophil activation involved in immune responses, focal adhesion, cell cycle, and p53 signaling pathway.

**Conclusion:**

PRGs could predict the prognosis of glioma and play significant roles in a tumor immune microenvironment.

## 1. Introduction

According to the data of Central Brain Tumor Registry of the United States, gliomas account for about 25.1% of all primary brain and other CNS tumors.^[1]^ They are currently treated with standard methods, including surgical resection, postoperative radiotherapy, and temozolomide concurrent chemotherapy.^[2]^ However, the prognosis of most patients with glioma is poor, especially those with glioblastoma (GBM), which is the most malignant glioma; furthermore, the overall 5-year survival rate of these patients is <10%.^[3]^ Clinically, we predict patients with glioma and create individualized treatment plans with biomarkers consisting of IDH1/2 mutation, MGMT methylation status, IDH status, and 1p/19q status.^[1]^ However, the prediction of the survival of patients with glioma is still inaccurate, so a novel reliable prognostic model should be developed.

Pyroptosis is a newly discovered inflammatory programmed cell death mainly characterized by cellular swelling, membrane perforation, cell content release, DNA breakage, and chromatin condensation.^[4,5]^ It is triggered by the activation of caspase-1/GSDMD or caspase-3/GSDME.^[6]^ It also elicits dual effects on tumors. On the one hand, pyroptosis-related inflammatory cytokines and pathways promote tumor growth and invasion.^[7,8]^ On the other hand, inducing pyroptosis directly inhibits tumor proliferation.^[6]^

Pyroptosis, protective autophagy, and apoptosis in GBM cells can be induced by galangin, which is a natural flavonoid. Galangin-induced pyroptosis and apoptosis are significantly enhanced through the inhibition of autophagy by 3-mA.^[9]^ Other studies have shown that the proinflammatory effect of pyroptosis is related to the regulation of a tumor immune microenvironment (TIME). The induction of pyroptosis of some tumor cells leads to the regression of T cell-dependent tumors, an increase in T cells, M1 macrophages, and NK cells, and a decrease in M2 macrophages, myeloid-derived suppressor cells, regulatory T cells, and neutrophils.^[10]^

Pyroptosis can facilitate cancer progression and antitumor processes. However, few studies have been performed on the functions of pyroptosis in gliomas. Therefore, this study was conducted to explore the prognostic value of PRGs between glioma tissues and adjacent normal tissues and the correlation between prognostic and TIME.

## 2. Methods

Since Bioinformatics does not involve the collection of private information, this research does not require ethical approval.

### 2.1. Datasets

RNA-seq transcriptome data and clinical information were downloaded from the TCGA data portal (https://portal.gdc.cancer.gov/) on August 6, 2021. A total of 698 glioma samples and 5 adjacent normal tissues were downloaded. The RNA-seq data and clinical data of the external validation cohort were downloaded from the GEO database (https://www.ncbi.nlm.nih.gov/geo/, ID: GSE43378).

### 2.2. Identification of differentially expressed PRGs

Fifty-two PRGs were extracted from published literatures, and they are presented in Table S1, Supplemental Content, http://links.lww.com/MD/G883, which illustrates 52 PRGs and full names. The expression data in datasets were normalized to fragment per kilobase million values before comparison. The “limma” package was used to identify differentially expressed PRGs with *P* = .05. The differentially expressed PRGs were notated as follows: * if *P* = .05, ** if *P* = .01, and *** if *P* = .001. A PPI network for the differentially expressed PRGs was applied with the Search Tool for the Retrieval of Interacting Genes (https://string-db.org/).

### 2.3. Construction and validation of the PRG prognostic model

Cox regression analysis was applied to estimate the relativity between survival status and every gene in the TCGA cohort and evaluate the prognostic value of PRGs. A total of 558 survival-related genes were identified by setting 0.001 as the cutoff *P* value for further analysis. Then, a LASSO Cox regression model was used to develop a prognostic model. Lastly, 16 genes and corresponding coefficients were retained, and the penalty parameters were determined in accordance with the minimum standard. The “scale” function in *R* was applied to calculate the risk score after the concentration and standardization of TCGA expression data. The TCGA patients with glioma were divided into low- and high-risk subgroups with a median risk score, and the OS time of the 2 subgroups was compared via Kaplan–Meier analysis. Principal component analysis (PCA) based on the 16-gene signature was implemented with the “prcomp” function in the “stats” *R* package. The “survival,” “time¬ROC,” and “survminer” packages were applied to execute 1-, 3-, and 5-year ROC curve analysis. A glioma cohort from the GEO database (GSE43378) was adopted for the validation study. The expression of each PRG was also standardized with the “scale” function. Then, the risk score was calculated with the same formula used in the TCGA cohort. Patients with glioma in the GSE43378 cohort were also stratified into 2 subgroups with the same median risk score from the TCGA cohort and compared to verify the gene model.

### 2.4. Independent prognostic analysis of the prognostic model

Clinical information (age, gender, and grade) was extracted from the TCGA and GEO cohorts. These variables were analyzed using univariate and multivariate Cox regression models combined with the risk score in our regression model.

### 2.5. Functional enrichment analysis of PRGs between the low- and high-risk groups

Patients with gliomas were divided into 2 subgroups based on the median risk score of the TCGA cohort. Differentially expressed PRGs between low- and high-risk groups were screened on the basis of specific criteria (|log2FC| ≥ 1 and FDR < 0.05). GO and KEGG analyses were implemented using a “clusterprofiler” package in terms of the differentially expressed PRGs. The fraction of infiltrating immune cells was calculated, and the immune-related pathways were estimated with the “gsva” package.

### 2.6. Statistical analysis

**Figure 1.**
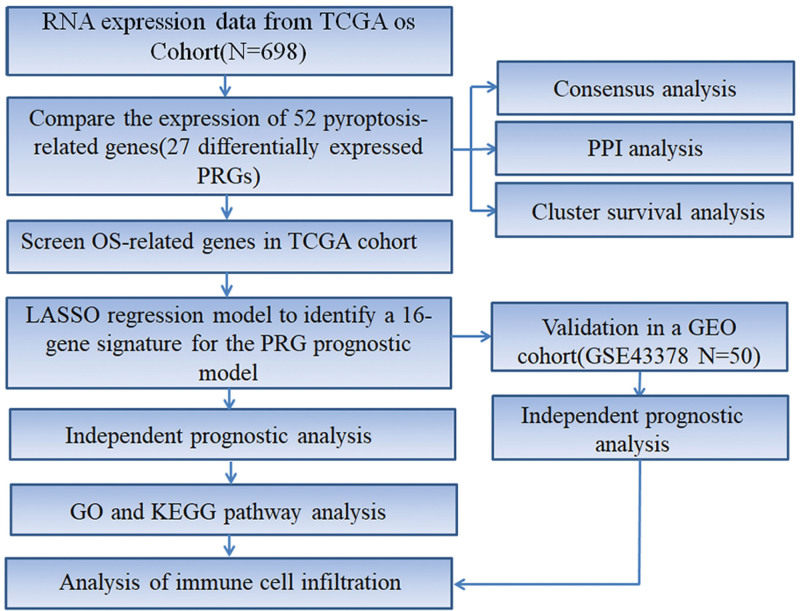
Workflow diagram. The specific workflow graph of data analysis.

One-way ANOVA was adopted to compare 2 subgroups, and Pearson correlation test was performed to analyze the correlation between subtypes, risk scores, and clinicopathological features. The Kaplan–Meier method was conducted to generate the survival curve, and the difference between the 2 groups was compared with the log-rank test. A Cox regression model was used for univariate and multivariate analyses to determine the independent prognostic value of risk scores combined with other clinical features. Mann–Whitney test was employed to compare immune cell infiltration and immune pathway activation between low- and high-risk groups. Data were statistically analyzed using *R* version 4.1.0 and SPSS 26.0 (IBM, NY). The overall flow chart is shown in Fig. 1.

## 3. Results

### 3.1. Identification of differentially PRGs between normal and tumor tissues

**Figure 2.**
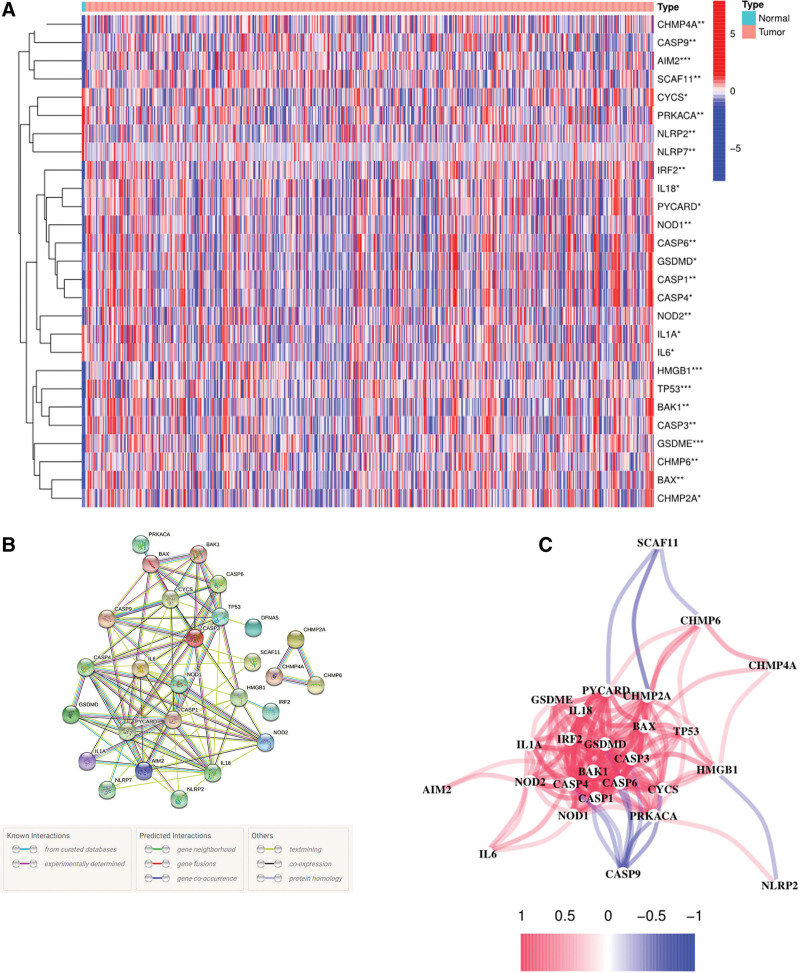
Expressions of the 27 PRGs and the interactions among them. (A) Heatmap (red represents high expression, whereas blue represents low expression) of the PRGs between the normal (N, brilliant blue) and the tumor tissues (T, red). *P* values were shown as: *P = .05; **P = .01; *** *P* = .001. B PPI network showing the interactions of the PRGs (interaction score = 0.9). (C) The correlation network of the PRGs (red line: positive correlation; blue line: negative correlation. The depth of the colors reflects the strength of the relevance).

The expression levels of 52 PRGs were compared from 5 normal and 698 tumor tissues in TCGA data, and 27 differentially expressed PRGs were identified (*P* = .01). Among them, 6 genes (CYCS, IL1A, IL6, PRKACA, NLRP2, and NLRP7) were downregulated, and 21 other genes (BAK1, BAX, CASP1, CASP3, CASP4, CHMP2A, CHMP4A, CHMP6, GSDMD, GSDME, HMGB1, IL18, IRF2, TP53, AIM2, CASP6, CASP9, NOD1, NOD2, SCAF11, and PYCARD) were enriched in the glioma group. The RNA levels of the genes are shown as heatmaps in Fig. 2A. Protein–protein interaction (PPI) analysis was performed to further explore the interaction of the PGRs (Fig. 2B). Then, 0.9 (the highest confidence) was set as the minimum interaction score required for PPI analysis, and our results indicated that CASP3, CASP4, CASP9, PYCARD, AIM2, NOD1, NOD2, BAX, CYCS, IL6, HMGB1, and TP53 were hub genes. These genes were the differentially expressed PRGs between normal and tumor tissues. The correlation network containing all PRGs is presented in Fig. 2C.

### 3.2. Tumor classification based on differentially expressed PRGs

**Figure 3.**
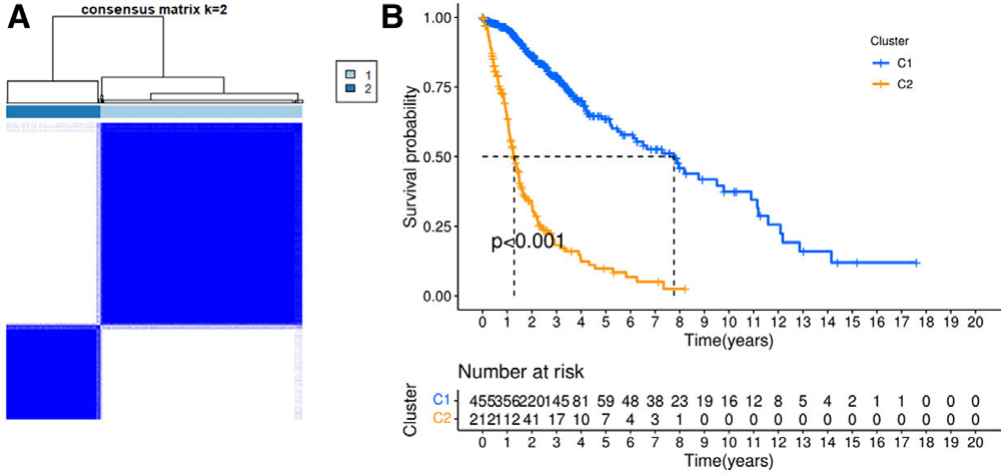
Tumour classification based on the differentially expressed PRGs. (A) 667 patients with glioma were grouped into 2 clusters according to the consensus clustering matrix for k = 2. (B) Kaplan-Meier OS curves for patients with glioma in 2 clusters (cluster1/2).

A consistent cluster analysis of all 667 patients with glioma and complete survival information in the TCGA cohort was carried out to explore the relationship between the expression of 27 differentially expressed PRGs and glioma subtypes. According to the similarity between the expression level of 27 differentially expressed PRGs and the proportion of fuzzy clustering test, k = 2 had the best clustering stability from k = 2–10 (Fig. 3A). A total of 667 patients with glioma were divided into 2 subtypes, namely, clusters 1 (n = 455) and 2 (n = 212). The OS time between clusters 1 and 2 was compared, and significant differences were found (*P* = .001; Fig. 3B).

### 3.3. Construction of a prognostic model in the TCGA cohort

**Figure 4.**
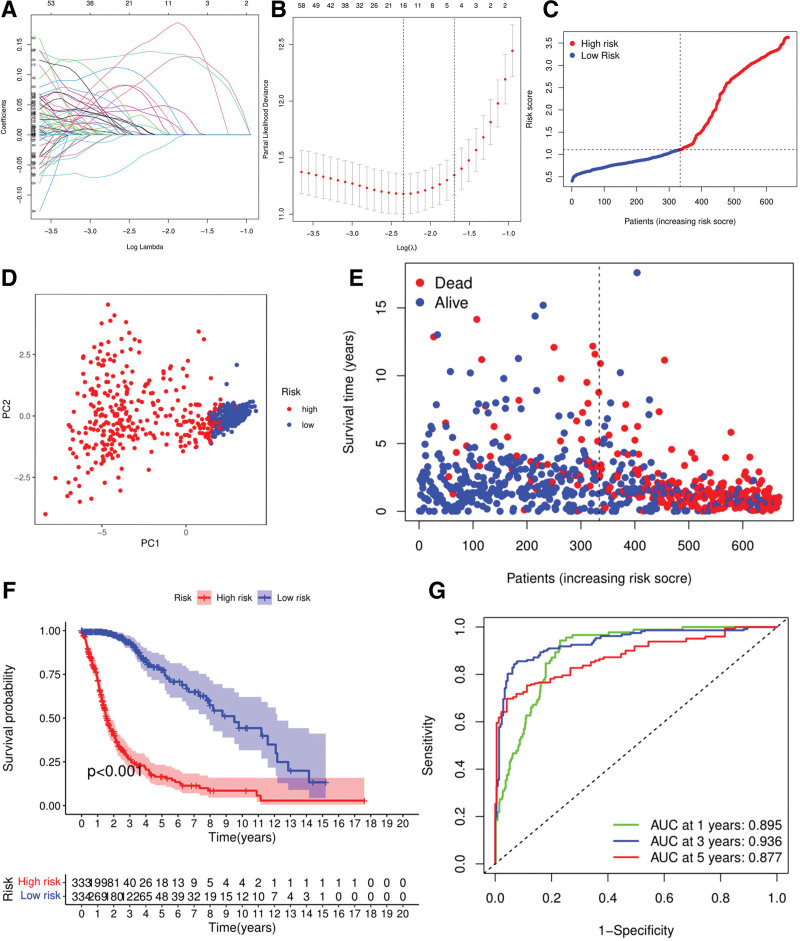
Construction of risk signature in the TCGA cohort. (A) LASSO regression of the 16 OS-related genes. (B) Cross-validation for tuning the parameter selection in the LASSO regression. (C) Distribution of patients based on the risk score. (D) PCA plot for patients with glioma based on the risk score. (E) The survival status for each patient (low-risk population: on the left side of the dotted line; high-risk population: on the right side of the dotted line). (F) Kaplan-Meier curves for the OS of patients in the high- and low-risk groups. (G) ROC curves demonstrated the predictive efficiency of the risk score.

Univariate Cox regression analysis was conducted to screen survival-related genes. The 558 genes that met the criteria of *P* = .01 were screened for further analysis. A LASSO Cox analysis was performed, and 16-gene signature was constructed on the basis of the optimal λ value (Fig. 4A and B). The risk score of the equation was calculated as follows: risk score = (0.150 × RAB42 exp.) + (0.039 × MSN exp.) + (0.071 × TOM1L1 exp.) + (0.089 × EMP3 exp.) + (0.034 × APOBEC3C exp.) − (0.035 × CRTAC1 exp.) + (0.013 × FBXO17 exp.) + (0.001 × HOXA2 exp.) + (0.001 × SNHG18 exp.) + (0.042 × STEAP3 exp.) + (0.077 × PLAUR exp.) + (0.137 × IGFBP2 exp.) + (0.002 × RARRES1 exp.) + (0.012 × LOXL1 exp.) + (0.099 × EN1 exp.) + (0.017 × FABP5 exp.). A total of 667 patients with glioma were separated into high- and low-risk subgroups according to the median score (Fig. 4C). PCA demonstrated that patients with different risks were divided into 2 clusters (Fig. 4D). Less deaths and longer survival time were found in the low-risk group than in the high-risk group (Fig. 4E). The OS time significantly differed between the 2 groups (*P* = .001; Fig. 4F). In the TCGA validation cohort, the 1-, 3-, and 5-year AUC values of the 16 risk signatures were 0.895, 0.936, and 0.877, respectively (Fig. 4G). These results indicated that the prognostic model had a stable and robust OS prediction capability for patients with glioma.

### 3.4. External validation of the risk signature

**Figure 5.**
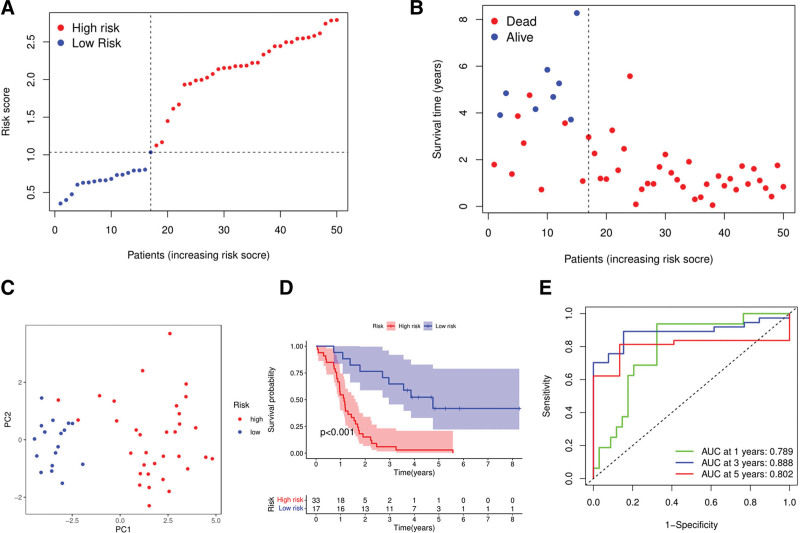
Validation of the risk model in the GEO cohort. (A) Distribution o f patients in the GEO cohort based on the median risk score in the TCGA cohort. (B) PCA plot for patients with glioma. (C) The survival status for each patient (low-risk population: on the left side of the dotted line, high-risk population: on the right side of the dotted line). (D) Kaplan-Meier curves for comparison of the OS between low- and high-risk groups. (E) Time-dependent ROC curves for glioma patients.

A total of 50 patients with gliomas from a Gene Expression Omnibus (GEO) cohort (GSE43378) were utilized as the validation set. Before further analysis, the gene expression data were normalized by the “scale” function. Based on the median risk score in the TCGA cohort, 17 patients in the GEO cohort were classified into the low-risk group, and the 33 other patients were classified into the high-risk group (Fig. 5A). PCA showed a satisfactory separation between the 2 subgroups (Fig. 5B). The survival times of the patients in the low-risk subgroup were longer than those in the high-risk subgroup, and the death rates of the former were lower than those of the latter (Fig. 5C). Kaplan–Meier analysis also indicated a significant difference in the survival rate between the low- and high-risk groups (*P* = .001; Fig. 5D). The ROC curve analysis of the GEO cohort showed that our model had good predictive efficacy (AUC = 0.789 for 1-year survival, 0.888 for 3-year survival, and 0.802 for 5-year survival; Fig. 5E).

### 3.5. Independent prognostic analysis of the risk model

**Figure 6.**
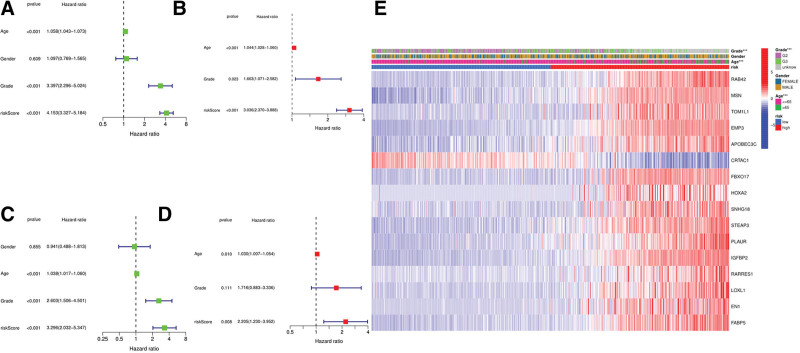
Univariate and multivariate Cox regression analyses for the risk score. (A) Univariate analysis for the TCGA cohort (age, grade and risk score were risk factors, *P* = .05). (B) Multivariate analysis for the TCGA cohort (age, grade and risk score were risk factors, *P* = .05). (C) Univariate analysis for the GEO cohort (age, grade and risk score were risk factors, *P* = .05). (D) Multivariate analysis for the GEO cohort (age and risk score were risk factors, *P* = .05). (E) Heatmap (blue: low expression; red: high expression) for the connections between, clinicopathologic features and the risk groups (***P = .001).

Univariate and multivariate Cox analyses were applied to assess whether the risk score is an independent prognostic factor of patients with glioma. Univariate Cox regression analysis showed that the risk score was an independent factor of the prediction of survival in TCGA and GEO cohorts (HR = 4.153, 95% CI: 3.327–5.184 and HR: 3.296, 95% CI: 2.032–5.347; Figs. 6A and C). Multivariate analysis also revealed that risk score was an independent prognostic factor (HR = 3.036, 95% CI: 2.370–3.888 and HR: 2.205, 95% CI: 1.230–3.952; Figs. 6B and D) for patients with gliomas in both cohorts. The heatmap of the clinical characteristics of the TCGA cohort (Fig. 6E) indicated that age and grade were distributed differently between high- and low-risk subgroups (*P* = .05).

### 3.6. Functional analyses with the risk model

**Figure 7.**
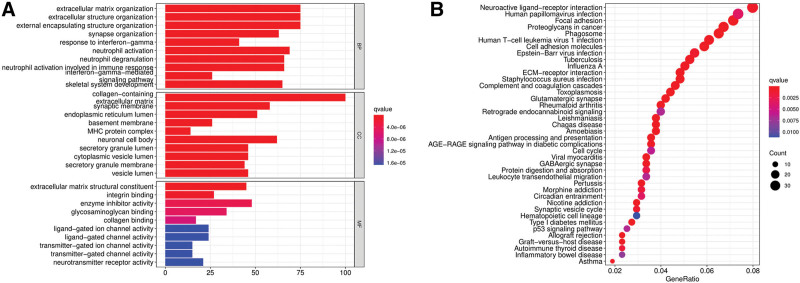
Functional analysis based on the differentially expressed PRGs between the 2-risk groups in the TCGA cohort. (A) Barplot graph for GO path ways (the longer bar means the more genes enriched, and the increasing depth of red means the differences were more obvious); (B) Bubble graph for KEGG enrichment (the bigger bubble means the more genes enriched, and the increasing depth of red means the differences were more obvious; q value: the adjusted *P* value).

The “limma” *R* package was used to extract differentially expressed PRGs by applying the criteria of FDR < 0.05 and | log2FC | ≥ 1 and further explore the differences in gene functions and pathways among subgroups classified according to the risk model. A total of 1016 differentially expressed PRGs were identified among the high- and low-risk groups in the TCGA cohort. Among them, 626 genes in the high-risk group were upregulated, and 390 genes were downregulated. Then, GO and KEGG pathway analyses were carried out on the basis of these differentially expressed PRGs. The results showed that the differentially expressed PRGs were mainly related to the neutrophil activation involved in immune responses, focal adhesion, cell cycle, and p53 signaling pathways (Figs. 7A and B).

### 3.7. Comparison of immune activities among subgroups

**Figure 8.**
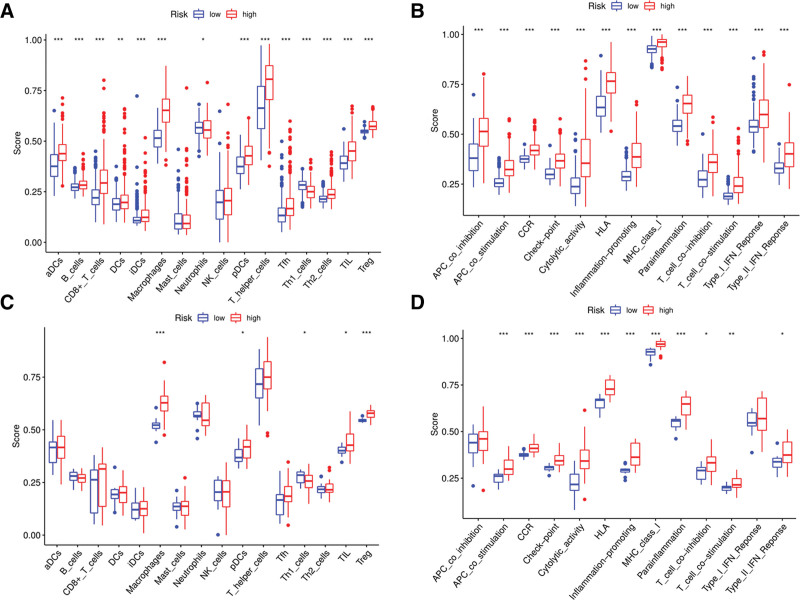
Comparison of the ssGSEA scores for immune cells and immune pathways. (A, B) Comparison of the enrichment scores of 16 types of immune cells and 13 immune-related path ways between low- (blue box) and high risk (red box) group in the TCGA cohort. (C, D) Comparison of the tumor immunity between low- (blue box) and high-risk (red box) group in the GEO cohort. *P* values were showed as: ns not significant; *P = .05; **P = .01; ***P = .001.

The enrichment scores of immune cells (16 types) and the activities of immune-related pathways (13 types) were compared on the basis of functional analysis by adopting the single-sample gene set enrichment analysis (ssGSEA) in TCGA and GEO cohorts. In the TCGA cohort (Fig. 8A), the population of protumor immune cells, especially regulatory T (Treg) cells, macrophages, and other immune cells, such as CD8 + T cells, T helper (Th) cells (Tfh and Th2 cells), tumor-infiltrating lymphocytes (TILs), and B cells, was higher in the high-risk group than in the low-risk group. The activity of 13 immune-related pathways was higher in the high-risk subgroup than in the low-risk subgroup in the TCGA cohort (Fig. 8B). Similar conclusions were obtained in the GEO cohort (Figs. 8C and D).

## 4. Discussion

Pyroptosis is a new kind of programmed cell death, which plays a dual role in tumor progression and treatment mechanism. In several tumors, novel PRG signatures have been identified to predict prognosis.^[11,12]^ However, the role of PRG in glioma has not been clarified, and our study aims to elucidate this role.

Our study produced a signature featuring 16 novel PRGs (RAB42, MSN, TOM1L1, EMP3, APOBEC3C, CRTAC1, FBXO17, HOXA2, SNHG18, STEAP3, PLAUR, IGFBP2, RARRES1, LOXL1, EN1, and FABP5) and found that it could be used for the prognosis of the OS of patients with glioma. The high expression of these genes was associated with poor survival outcomes except for CRTAC1 possibly because of its negative regulation of pyroptosis. Among the risk-associated genes, RAB42 (protein-coding gene), as a member of the RAS oncogene family, is involved in protein metabolism and RAB geranylgeranylation.^[13]^Some scholars believed that RAB42 is significantly associated with the prognosis of glioma via bioinformatic analysis.^[14]^ Moesin (MSN) expression is upregulated in glioblastoma cells because of the increase in cell proliferation, invasion, and migration.^[14]^ miR-200c can inhibit the tumor progression of glioma by targeting MSN.^[15]^ In our study, RAB42 and MSN also acted as cancer-promoting genes because of their negative correlation with survival time. MYB1-like protein 1 (TOM1L1) is relevant to bone metastasis in breast cancer. TOM1L1 amplification may enhance the metastasis of ERBB2-positive breast cancer.^[16]^ EMP3 has carcinogenic properties in high-grade gliomas (HGG), and its overexpression may predict poor clinical prognosis in glioblastoma (GBM).^[17]^ However, EMP3 is a tumor-suppressor gene of low-grade glioma (LGG).^[18]^ In our present study, a high EMP3 expression was associated with poor survival, confirming that EMP3 is an oncogene. Studies have shown that the APOBEC3C expression positively affects the invasiveness and prognosis of breast cancer, glioma, hepatocellular carcinoma, and prostate cancer.^[19,20]^ FBXO17 overexpression can accelerate the proliferation, migration, and invasion of glioma cells via the Akt/GSK-3β/Snail pathway.^[21]^ FBXO17 is a new and robust marker for predicting survival in patients with HGG.^[22]^ HOXA2, which is expressed highly in patients with glioma, is an independent factor of poor prognosis.^[23]^ lncRNA SNHG18 has been shown to regulate epithelial–mesenchymal transformation and cytoskeleton remodeling and enhance the radiation resistance of glioma cells in vitro and in vivo.^[24]^ STEAP3 is a direct target gene of p53, which regulates the G2–M phase transition to accelerate cell proliferation. STEAP3 is a potential prognostic biomarker and therapeutic target of GBM.^[25]^ PLAUR can encode the urokinase receptor, which can promote cell migration, be used to evaluate the prognosis of glioma,^[26]^ and regulate apoptosis in polyautoimmunity.^[27]^ IGFBP2 is expressed highly in GBM and involved in the regulation of the Oct4 transcript, thereby inducing the stemness of glioma cells.^[28]^ RARRES1 is a retinoic acid receptor-response gene, which is dysregulated in a variety of cancers, such as nasopharyngeal carcinoma, colorectal adenocarcinoma, and melanoma.^[29]^ RARRES1, which is considered a novel immune-related biomarker, can regulate the proliferation and migration of tumor cells and may be used as an oncogene of GBM.^[30]^ LOXL1 is a member of the lysyl oxidase (LOX) family and can play an antiapoptotic capacity by Wnt/β-catenin signaling and become a biomarker to guide the clinical treatment of glioma.^[31]^ Engrailed 1 (EN1) may accelerate the proliferation, migration, and multinucleation of cancer cells and is highly expressed in tumors.^[32]^ EN1 overexpression is a poor prognostic biomarker in quintuple-negative breast cancer.^[33]^ Fatty acid binding protein 5 (FABP5), as a member of the FABP protein family, is closely related to the occurrence, development, and metastasis of a variety of tumors.^[34]^ The knockdown of FABP5 that plays an oncogene role in glioma significantly reduces the proliferation, migration, and invasion.^[35]^ Regarding the protective gene, cartilage acidic protein 1 (CRTAC1), which is inhibited, can reduce apoptosis induced by ultraviolet B irradiation. CRTAC1 downregulation attenuates the UVB-induced pyroptosis of epithelial cells in human lens.^[36]^ The high expression of CRTAC1 prolongs the survival time of patients with LGG.^[37]^ Similarly, our finding indicated that it acts as a tumor suppressor. In summary, 16 genes in the prognostic model were treated as “potential” PRGs. Our results provided a theoretical basis for describing new pyroptosis-related genes. However, the relationship between gene-mediated pyroptosis and tumor development remains unknown. In addition to CRTAC1 as a tumor suppressor gene, other genes could have a pro-oncogenic function in patients with glioma.

Our analysis on the differentially expressed PRGs between different risk groups revealed that the PRGs were mainly involved in the neutrophil activation involved in immune responses, focal adhesion, cell cycle, and p53 signaling pathway. Our GO and KEGG analysis results suggested that pyroptosis could regulate the composition of TIME. Glioma cells can secrete immunomodulatory factors and attract a few kinds of cells, including immunocytes, into the tumor microenvironment (TME).^[38]^ A previous study showed that the diverse content of glioma immune cells is related to varying prognosis.^[39]^ Glioma-associated microglia and macrophages, together with neutrophils and dendritic cells, constitute a glioma TME to regulate and inhibit tumor immune responses.^[40]^ In our study, more immune cells infiltrated high-risk gliomas. The levels of most immune cells, including Tregs and antitumor immune cells,^[41]^ significantly increased in high-risk glioma. Although the increase in antitumor immunocytes seems contradictory in high-risk glioma, the infiltration of protumor immune cells might offset the infiltration of antitumor immunocytes and destroy the balance between the 2 kinds of cells. Furthermore, our results suggested that the infiltration of B cells, CD8 + T cells, dendritic cells, macrophages, and Tregs was correlated with poor prognosis. Consequently, the diversity of immune-related cell components might be a key factor of the different survival times between the 2 risk types.

Macrophages are the main cell type of the immune system and key drivers of inflammation.^[40]^ In tumor progression, macrophages can establish an inflammatory environment, stimulate tumor angiogenesis, enhance the invasion and migration of cancer, and inhibit antitumor immunity.^[42]^ The increase in tumor-associated macrophages is related to poor clinical prognosis.^[43]^ Dendritic cells, as antigen-presenting cells, are essential for T cell-mediated immunity and tumor immunity activation.^[44]^ However, they are often insufficient to induce an effective immune response in tumors.^[45]^ In addition, they can be used by tumor cells to evade immunity in a TME.^[46]^ Tregs can inhibit antitumor immunity and is associated with poor clinical results,^[47]^ which is consistent with our results. B cells are important cellular components of human immunity and implicated in biological functions, such as antibody secretion, antigen presentation, and T cell activation.^[48]^ B cells are involved in tumor growth and proliferation.^[49]^ In addition, neutrophils are critical effector cells in our immune system. Their effect on glioma has 2 aspects, which are mainly determined by the state of maturation and activation. Immunosuppression induced by circulating neutrophils can promote tumor growth by secreting arginase I.^[19]^ However, the activation of neutrophils elicits antitumor effects through antibody-dependent cytotoxicity.^[50]^ These findings are consistent with our conclusions.

In conclusion, our study showed that pyroptosis was closely related to glioma because most PRGs were expressed differentially between normal and glioma tissues. An independent prognostic model related to PRGs was established to predict the prognosis of patients with glioma in TCGA and GEO cohorts. Our study provided a new novel gene signature for predicting the prognosis of patients with glioma and an important basis for exploring PRGs and immunotherapy. However, this study had some limitations. The prognosis model included 16 prognosis-related PRGs, which were not succinct and effective. As such, future research will focus on building a more succinct and stable prognostic model.

## Acknowledgment

The authors thank all the researchers who supported The Cancer Genome Atlas.

## Author contributions

Study design and supervision were performed by XDZ, ZGY, and JQ contributed the collection, analysis, and interpretation of data. SYX planned and wrote the manuscript. FRZ and YCL critically revised the manuscript. All authors gave approval for this version of the manuscript to be published and agree to be accountable for all aspects of the work.

## Supplementary Material


