# Supplementary material for: Identification of a pyroptosis-related prognosis gene signature and its relationship with an immune microenvironment in gliomas
Source: Medicine (Baltimore). 2022 Jul 15;101(28):e29391. doi: 10.1097/MD.0000000000029391 (PMC11132325; doi:10.1097/MD.0000000000029391)
Supplement: Supplementary file 1 [file medi-101-e29391-s001.pdf]

Table S1

Fifty-two PRGs were extracted from published literatures

Genes	Full-names
BAK1	BCL2 antagonist/killer 1
BAX	BCL2 associated X
CASP1	cysteine-aspartic acid protease-1
CASP3	cysteine-aspartic acid protease-3
CASP4	cysteine-aspartic acid protease-4
CASP5	cysteine-aspartic acid protease-5
CHMP2A	charged multivesicular body protein 2A
CHMP2B	charged multivesicular body protein 2B
CHMP3	charged multivesicular body protein 3
CHMP4A	charged multivesicular body protein 4A
CHMP4B	charged multivesicular body protein 4B
CHMP4C	charged multivesicular body protein 4C
CHMP6	charged multivesicular body protein 6
CHMP7	charged multivesicular body protein 7
CYCS	cytochrome c, somatic
ELANE	elastase, neutrophil expressed
GSDMD	gasdermin D
GSDME	gasdermin E
GZMB	granzyme B
HMGB1	high mobility group box 1
IL18	interleukin 18
IL1A	interleukin 1 alpha
IL1B	interleukin 1 beta
IRF1	interferon regulatory factor 1
IRF2	interferon regulatory factor 2
TP53	tumor protein p53
TP63	tumor protein p63
AIM2	Absent in melanoma 2
CASP6	caspase 6
CASP8	caspase 8
CASP9	caspase 9
GPX4	glutathione peroxidase 4
GSDMA	gasdermin A
GSDMB	gasdermin B
GSDMC	gasdermin C
IL6	interleukin 6
NLRC4	NLR family CARD domain containing 4
NLRP1	NLR family pyrin domain containing 1
NLRP2	NLR family pyrin domain containing 2
NLRP3	NLR family pyrin domain containing 3
NLRP6	NLR family pyrin domain containing 6

NLRP7	NLR family pyrin domain containing 7
NOD1	nucleotide binding oligomerization domain containing 1
NOD2	nucleotide binding oligomerization domain containing 2
PJVK	pejvakin/deafness, autosomal recessive 59
PLCG1	phospholipase C gamma 1
PRKACA	protein kinase cAMP-activated catalytic subunit alpha
PYCARD	PYD and CARD domain containing
SCAF11	SR-related CTD associated factor 11
TIRAP	TIR domain containing adaptor protein
TNF	tumor necrosis factor
GZMA	granzyme A
